# RNA-seq and phytohormone analysis reveals the culm color variation of *Bambusa oldhamii* Munro

**DOI:** 10.7717/peerj.12796

**Published:** 2022-01-13

**Authors:** Yulian Jiao, Hu Zeng, Haitao Xia, Yueying Wang, Jinwang Wang, Chuan Jin

**Affiliations:** 1 Zhejiang Institute of Subtropical Crops, Zhejiang Academy of Agricultural Sciences, Wenzhou, Zhejiang, China; 2 Zhejiang Provincial Collaborative Innovation Center for Bamboo Resources and High-efficiency Utilization, Lin’an, Zhejiang, China; 3 Wuhan Metware Biotechnology Co., Ltd, Wuhan, China

**Keywords:** *Bambusa oldhamii*, Culm color variation, Phytohormone, Transcription factors, MYB, HY5, ABA, GA_1_, GA_7_, RNA-seq

## Abstract

**Background:**

The clumping bamboo *Bambusa oldhamii* Munro, known as “green bamboo”, is famous for its edible bamboo shoots and fast-growing timber. The green and yellow striped-culm *B. oldhamii* variety, named *B. oldhamii* f. *revoluta* W.T. Lin & J. Y. Lin, is an attractive system for researching the culm color variation of *B. oldhamii*.

**Methods:**

Millions of clean reads were generated and assembled into 604,900 transcripts, and 383,278 unigenes were acquired with RNA-seq technology. The quantification of ABA, IAA, JA, GA_1_, GA_3_, GA_4_, and GA_7_ was performed using HPLC–MS/MS platforms.

**Results:**

Differential expression analysis showed that 449 unigenes were differentially expressed genes (DEGs), among which 190 DEGs were downregulated and 259 DEGs were upregulated in *B. oldhamii* f. *revoluta*. Phytohormone contents, especially GA_1_ and GA_7,_ were higher in *B. oldhamii.* Approximately 21 transcription factors (TFs) were differentially expressed between the two groups: the bZIP, MYB, and NF-YA transcription factor families had the most DEGs, indicating that those TFs play important roles in *B. oldhamii* culm color variation. RNA-seq data were confirmed by quantitative RT-PCR analysis of the selected genes; moreover, phytohormone contents, especially those of ABA, GA_1_ and GA_7_, were differentially accumulated between the groups. Our study provides a basal gene expression and phytohormone analysis of *B. oldhamii* culm color variation, which could provide a solid fundamental theory for investigating bamboo culm color variation.

## Introduction

Bamboo belongs to the Poaceae subfamily Bambusoideae, comprises over 1,600 bamboo species, and is extensively distributed in tropical and subtropical regions, such as Africa, South America, and South Asia (Vorontsova et al., 2016). Owing to its rapid growth, bamboo is widely used as a material in the biofuels, charcoal, timber, craft, furniture, house building, and paper-making industries (Ramakrishnan et al., 2020). Based on the rhizome structure and expansion characteristics of the plant, bamboos are divided into two major classifications: clumping bamboos (pachymorph or sympodial), such as *Dendrocalamus latiflorus*, *Bambusa oldhamii*, and *Bambusa chungii*; and running bamboos (leptomorph or monopodial), such as *Phyllostachys edulis* (Ramakrishnan et al., 2020), *Phyllostachys vivax*, and *Phyllostachys violascens* (Lieurance et al., 2018).

*B. oldhamii* is a species of clumping bamboo known as “green bamboo”; it is generally distributed in the southeast of China and is famous for its delicious edible rhizome buds (bamboo shoots, locally called ‘MaTiSun’) and fast-growing culm timber (Wu et al., 2009; Lieurance et al., 2018). *B. oldhamii* is characterized by its clumping rhizome and entirely green culm; however, a natural variety of *B. oldhamii*, named *B. oldhamii* f. *revoluta* W.T. Lin & J. Y. Lin, has green and yellow striped culms. The amazing culm color variation of *B. oldhamii* is of great interest. Research on color variation in bamboo is rare, but such studies have been widely conducted on colorful trees and flowers, such as blue-petal water lily (*Nymphaea colorata*) (Zhang et al., 2020), *Paeonia suffruticosa* (Lv et al., 2020), and *Rosa chinensis* (Raymond et al., 2018).

Chlorophyll, carotenoids, anthocyanins, and betalains are major pigments that contribute to plant colors (Grotewold, 2006). Chlorophyll can absorb sunlight for plant photosynthesis and it is responsible for the green color of almost all green plants (Hörtensteiner, 2009). Carotenoids are liposoluble and produce red, orange, and yellow pigments in photoautotrophic organs in plants (Stanley & Yuan, 2019). Anthocyanins are water soluble and less stable than carotenoids, and they cause flower and fruit colors ranging from shiny orange to pink and red to blue (Castañeda Ovando et al., 2009). Betalains are water soluble and indole-derived glycoside pigments discovered in the Caryophyllales order and *Basidiomycota phylum*; they comprise red to red-violet betacyanins and yellow-orange betaxanthins (Azeredo, 2009).

The plant hormones abscisic acid (ABA) and jasmonic acid (JA) could promote anthocyanin biosynthesis, while auxin and gibberellin (GA) could inhibit anthocyanin biosynthesis (Jaakola, 2013). Abscisic acid (ABA) treatment can increase anthocyanin accumulation in berry peels, and upregulate the expression of *MYB113*-like, *bZIP42*-like, and *UGT85A2*-like genes (Saito et al., 2018). JA treatment could increase chlorophyll and carotenoid accumulation (Poonam, Kaur & Geetika, 2013). JA treatment causes senescence symptoms with visible yellowing in Arabidopsis (He et al., 2002). The accumulation of anthocyanins is suppressed by auxin (Jeong et al., 2004). The plant hormone gibberellin (GA) can influence plant growth, germination, elongation, and flower development (Schwechheimer, 2012). GA_3_ increased vegetative growth and delayed fruit ripening time (Zang et al., 2016). Treatment with ABA and GA_3_ could increase the content of total chlorophyll, chlorophyll a, chlorophyll b, and carotenoids (Gomathinayagam et al., 2009).

The colorful flowers and fruits of the majority of plants are generated from the accumulation and balance of chlorophyll, carotenoids, and anthocyanins, even though each pigment has unique biosynthesis, regulation, and degradation pathways. Anthocyanins are flavonoids that play multiple roles in plant environmental stress responses, plant development, and food additives (Winkel-Shirley, 2001). The clade of R2R3-MYB transcription factors can increase anthocyanin production in tobacco and apples (Allan, Hellens & Laing, 2008). In apples, a cold-induced bHLH transcription factor, MdHLH3, can interact with its MYB partner to regulate the expression of anthocyanin biosynthesis genes *MdDFR* and *MdUFGT* and influence fruit coloration (Xie et al., 2012). MBW (MYB-bHLH-WDR) complexes can control flavonoid biosynthesis by regulating late biosynthetic gene expression (Xu, Dubos & Lepiniec, 2015).

The genome size of *B. oldhamii* was measured (Zhou et al., 2017), and the chloroplast genome size was found to be 139,350 bp (Wu et al., 2009). Phenylalanine ammonia-lyase (PAL) is a key enzyme in phenylalanine metabolism, phenylpropanoid biosynthesis, metabolic pathways, and the biosynthesis of secondary metabolites, and PAL influences the biosynthesis of lignins, alkaloids, flavonoids, and anthocyanins. The *BoPAL* gene was isolated from *B. oldhamii* and it has similar biochemical properties to those of PALs from other plants (Hsieh et al., 2010a; Hsieh et al., 2010b; Hsieh et al., 2011). RNA-seq technology changed the method of studying the transcriptome and exploring gene structure and expression (Shendure, 2008; McIntyre et al., 2011). In this paper, RNA-seq technology was applied to investigate the culm color variation of *B. oldhamii*. Key differentially expressed genes and transcription factors were discovered by comparing culm skin samples between *B. oldhamii* and *B. oldhamii* f. *revoluta*. Our results provide scientific and theoretical implications for understanding bamboo culm color variations.

## Materials and Methods

### Plant materials

The middle and lower culm internode epidermis samples that were removed from *B. oldhamii* (LZ) were labeled LZ_1, LZ_2, and LZ_3, and those from *B. oldhamii* f. *revoluta* W.T. Lin & J. Y. Lin (HLZ) were labeled HLZ_1, HLZ_2, and HLZ_3, representing three biological replicates of each type of bamboo. The culm skin samples were frozen in liquid nitrogen immediately for further phytohormone detection, RNA-seq, and relative gene expression. Total RNA was isolated using plant RNA isolation kits (Tiangen Biotech, Beijing, China).

### Library construction and sequencing

Library construction and sequencing steps were performed based on the Illumina HiSeq platform for RNA-seq protocols (https://www.illumina.com) at Wuhan Metware Biotechnology Co., Ltd. (Wuhan, China). The output data contained raw reads in fastq format, that were then processed for quality control, including filtering and trimming of low confidence bases, biased nucleotide composition, adapters, duplicates and low-quality reads to acquire clean reads. Trinity (2.6.6) (Grabherr et al., 2011) and Corset (1.07) (Davidson & Oshlack, 2014) were used to assemble the clean data and process the transcript cluster analysis, and the longest transcripts in each cluster were filtered out as unigenes. All of the following analyses were performed using unigene sequences.

### Gene annotation

Nr (NCBI nonredundant protein sequences), Pfam (Protein family), KOG (Protein family), Swiss-Prot, Trembl, KEGG (Kyoto Encyclopedia of Genes and Genomes), and GO (Gene Ontology) were used for gene annotation. The Nr, KOG, Swiss-Prot, Trembl, KEGG, and GO annotations were performed using BLAST (v2.7.1) with an *e*-value = 1e−5, and the Pfam annotation was performed using the hmmscan command of the HMMER 3.2 package with *e*-value = 0.01. Transcription factor annotation was performed with iTAK (1.7a) (Zheng et al., 2016) with default parameters.

### Gene expression analysis

We used the assembled transcriptome of Trinity as a reference and then mapped the clean reads of each sample to the reference with RSEM software. FPKM (Fragments Per Kilobase of transcript per Million fragments mapped) values were calculated to estimate gene expression and abundance after normalization of the mapped reads and transcript lengths. The R package Pheatmap was used to draw a heatmap with normalized log2(FPKM+1) data and clusters of expression patterns with kmeans_k = 10. The color from red to blue indicates gene expression from high to low.

### Differential expression analysis

After acquiring the abundance information and performing normalization, gene expression between the groups was compared. DESeq2 (1.22.2) was used to calculate the differentially expressed genes between the LZ and HLZ groups, which were corrected with FDR (False Discovery Rate) by Benjamini–Hochberg methods. Differentially expressed genes were filtered with the condition of —log2(Fold Change)— ≥ 1 and FDR < 0.05.

### Validation of RNA-seq analysis via qRT-PCR

The RNA-seq results were validated for selected genes using qRT-PCR assays. cDNA was synthesized with HiScript^®^ II Q RT SuperMix for qPCR (Vazyme, China). Quantitative Real-Time PCR (qRT-PCR) was performed on a LightCycler^®^ 480 II Real-Time PCR system (Roche International Diagnostics system, Switzerland) using the Unique Aptamer™ qPCR SYBR^®^ Green Master Mix. The components of the qRT-PCR were as follows: SYBR Premix Ex Taq (2x) (10 µl), forward primers (0.5 µM), reverse primers (0.5 µM), cDNA template (2 µl), and ddH_2_O to 20 µl. Then, qRT-PCR was performed as follows: initial denaturation at 95 °C for 5 min; 40 cycles of denaturation at 95 °C for 10 s and annealing at 72 °C; and finally, steps for melt-curve analysis (95 °C for 15 s, 60 °C for 60 s, 95 °C for 15 s). Actin was used as the internal control (Zeng et al., 2015; Table S1), and relative expression was calculated with the 2^−ΔΔCT^ method (Livak & Schmittgen, 2001).

### Detection of phytohormone contents

The quantification of endogenous abscisic acid (ABA), auxin (indole-3-acetic acid, IAA), jasmonic acid (JA), and gibberellic acid (GA_1_, GA_3_, GA_4_, and GA_7_) was performed by Genepioneer Biotechnologies Co., Ltd. (Nanjing, China) using an HPLC–MS/MS platform.

### Statistical analysis

The enrichment of up- and downregulated genes was determined using GOseq and KOBAS (Mao et al., 2005). The cor.test function was used to calculate the correlation between phytohormone contents and gene expression within the corresponding periods. Bar chart data of the phytohormone contents are reported as the mean ± SEM (*n* = 9) with a significant difference (*p* < 0.05) according to unpaired *t*-tests.

## Results

### Plant materials

**Figure 1:**
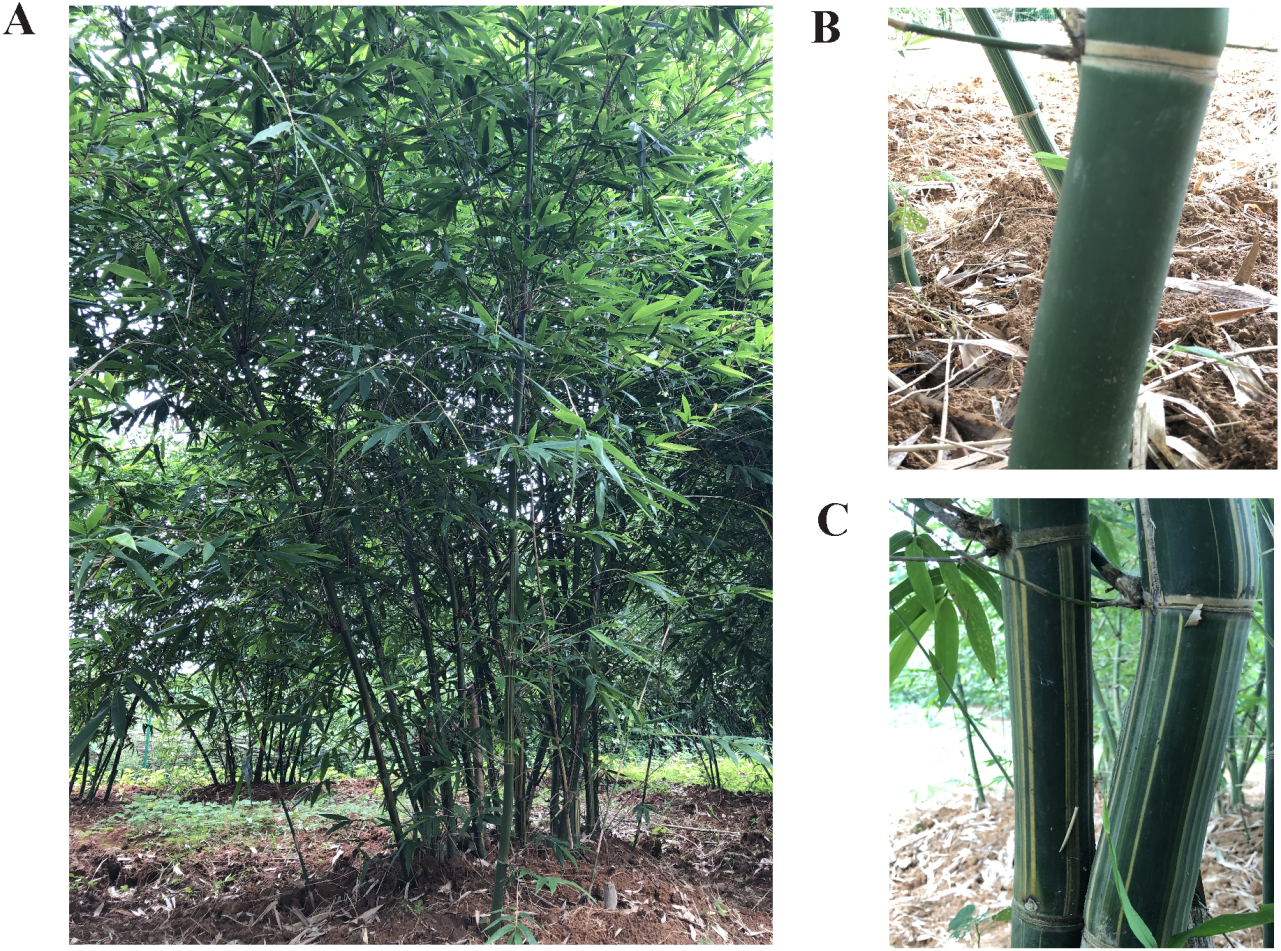
The bamboo of *Bambusa oldhamii.* (A) A clumping forest of *Bambusa oldhamii*, (B) an entirely green culm of *Bambusa oldhamii* (LZ), (C) a green culms with yellow stripes of *Bambusa oldhamii* f. *revoluta* (HLZ).

*B. oldhamii* (LZ) is a species of clumping bamboo (Fig. 1) and it has entirely green culms. The *B. oldhamii* variety referred to as *B. oldhamii* f. *reboluta* W.T. Lin & J. Y. Lin (HLZ) has green culms with yellow stripes of random widths. The culm skin was removed from LZ and HLZ to research the correlation of culm color variation on the phytohormone contents and gene expression levels.

### Transcriptome sequences and data output

Total RNA was isolated from the culm epidermis samples of *B. oldhamii* with three biological replicates marked LZ_1 - LZ_3 and from *B. oldhamii* f. *revoluta* with three biological replicates marked HLZ_1 - HLZ_3. After the cDNA library was constructed and sequenced, approximately 282 million raw sequence reads were obtained from the RNA-seq experiment, and 267 million clean sequence reads remained after filtering with a Q20 above 98% after quality control was performed. The error correction and GC content are shown as follows (Table 1).

**Table 1:** The read statistics of the data output and quality control.

**Sample**	**Raw reads**	**Clean reads**	**Clean base(G)**	**Error rate(%)**	**Q20(%)**	**Q30(%)**	**GC content(%)**
HLZ_1	51366578	47374034	7.11	0.02	98.56	95.33	51.4
HLZ_2	48793904	46491202	6.97	0.02	98.46	95.15	52.13
HLZ_3	44892558	43124866	6.47	0.02	98.57	95.4	52.72
LZ_1	48657910	46863938	7.03	0.02	98.4	94.99	52.72
LZ_2	46541436	43510176	6.53	0.02	98.47	95.22	52.19
LZ_3	42360436	40385740	6.06	0.02	98.63	95.6	52.29

### *De novo* assembly

The clean data were used for de novo assembly with Trinity (Grabherr et al., 2011), and overall, 604,900 transcripts and 383,278 unigenes were generated (Table 2). The average length of the transcripts was 670 bp, with an N50 of 1,033 bp and an N90 of 264 bp. Most (80%) of the transcripts were between 200 and 1,000 bp, while the remaining 20% of the transcripts had a length longer than 1,000 bp. The average length of the unigenes was 906 bp, with an N50 of 1,221 bp and an N90 of 433 bp. About 70% of the unigenes were shorter than 1,000 bp, while the remaining 30% of the unigenes were longer than 1,000 bp.

**Table 2:** Statistics of assembly transcripts and unigenes.

Type	Number	Mean length	N50	N90	Total Bases
Transcript	604,900	670	1,033	264	405,044,455
Unigene	383,278	906	1,221	433	347,398,391

### Gene annotation and functional classification

All of the unigenes were annotated using seven databases (Table 3) (https://doi.org/10.6084/m9.figshare.16912324). The annotation results produced 38.64%, 70.18%, 44.92%, 69.23%, 40.03%, 57.24%, and 47.26% unigenes annotated in the KEGG, NR, SwissProt, Trembl, KOG, GO, and Pfam databases, respectively. Approximately 274,681 unigenes were annotated in at least one database.

**Table 3:** Unigenes annotation statistics across seven databases.

Database	Number of genes	Percentage (%)
KEGG	148104	38.64
NR	268986	70.18
SwissProt	172168	44.92
Trembl	265339	69.23
KOG	153428	40.03
GO	219395	57.24
Pfam	181140	47.26
Annotated in at least one Database	274681	71.67
Total Unigenes	383278	100

**Figure 2:**
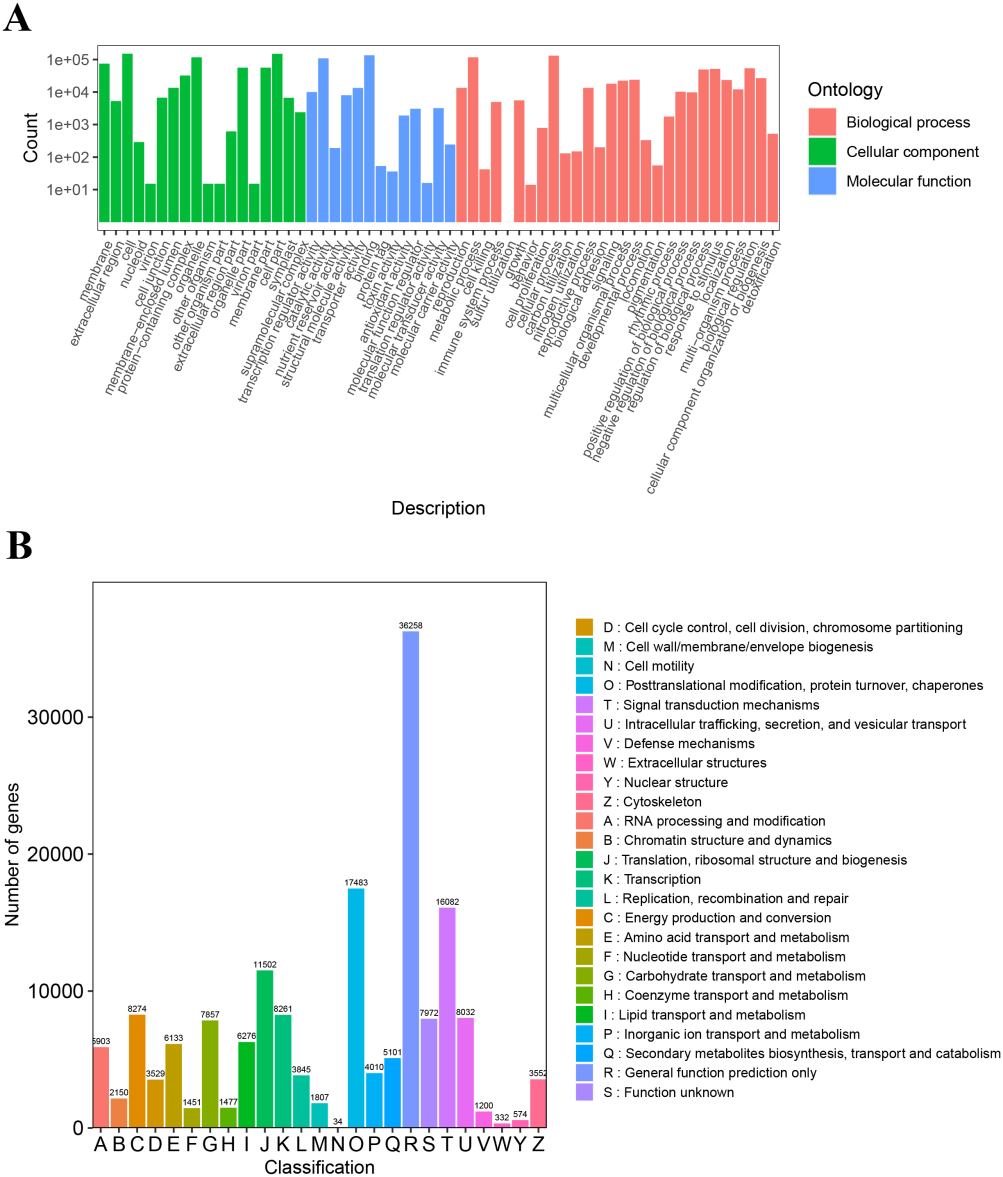
The GO and KOG functional classification of all unigenes. (A) The GO functional classification, (B) the KOG functional classification.

The unigenes annotated with GO functions were assigned to three main ontologies: molecular function (MF), cellular component (CC), and biological process (BP). The terms cellular process (GO:0009987), metabolic process (GO:0008152), biological regulation (GO:0065007), and response to stimulus (GO:0050896) were the most common BP ontologies; the terms cell (GO:0005623), cell part (GO:0044464), and organelle (GO:0043226) were the most common CC ontologies; and binding (GO:0005488), catalytic activity (GO: 0003824), transporter activity (GO:0005215), and transcription regulator activity (GO:0140110) were the most common MF ontologies (Fig. 2A; Table S2). The unigenes with KOG annotation were categorized as posttranslational modification, protein turnover, and chaperones; signal transduction mechanisms; translation, ribosomal structure and biogenesis; energy production and conversion; transcription; intracellular trafficking, secretion, and vesicular transport (Fig. 2B; Table S3). The majority of the annotated genes were characterized as ribosome pathway (ko03010) (1,841), glyoxylate and dicarboxylate metabolism (ko00630) (1,778), metabolic pathways (ko01100) (1,774), biosynthesis of secondary metabolites (ko01110) (1,768), oxidative phosphorylation (ko00190) (1,764), phenylpropanoid biosynthesis (ko00940) (1,755), carbon metabolism (ko01200) (1,742), glycolysis/gluconeogenesis (ko00010) (1,723), MAPK signaling pathway-plant (ko04016) (1,720), biosynthesis of amino acids (ko01230) (1,714), and pyruvate metabolism (ko00620) (1,710) (Table S4).

### Expression patterns of differentially expressed genes (DEGs)

**Figure 3:**
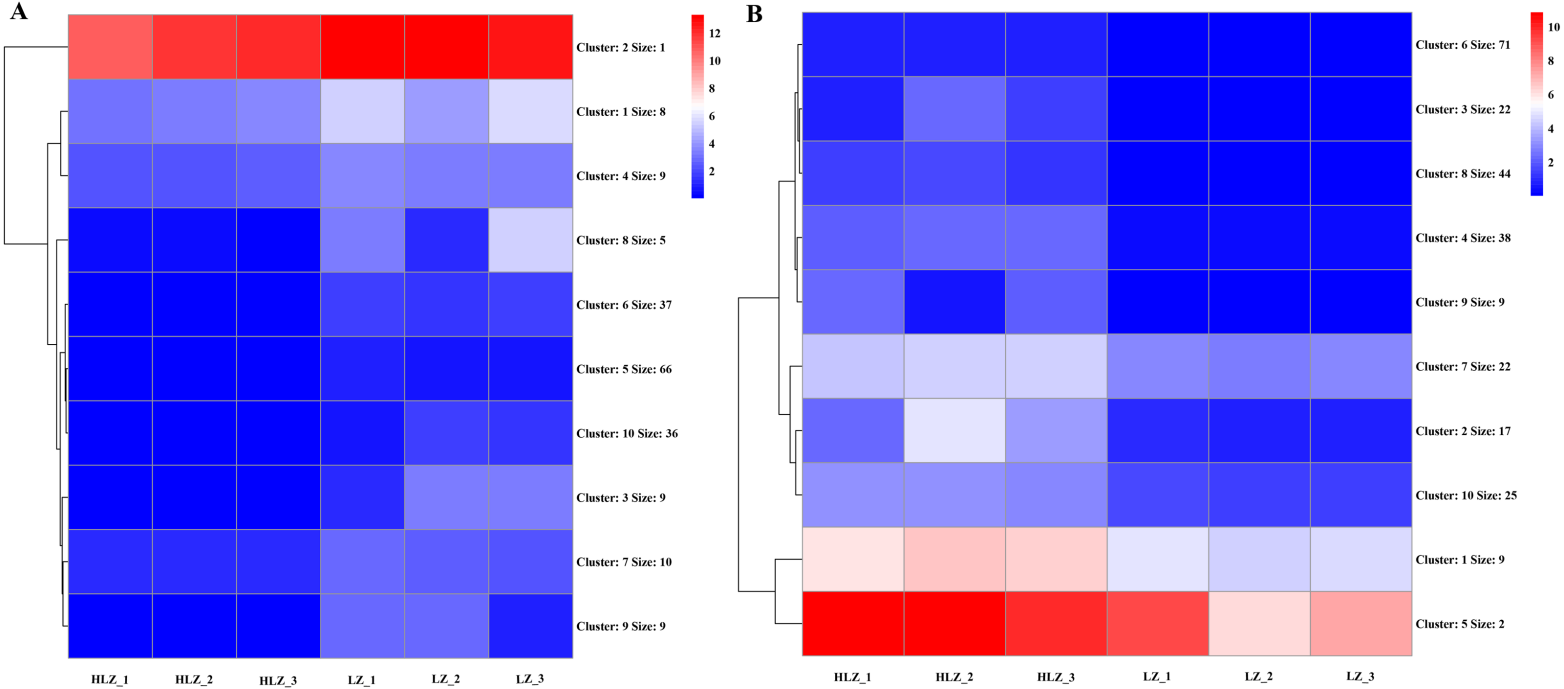
The cluster expression patterns of DEGs between HLZ and LZ. (A) The clusters expression patterns of downregulated DEGs in HLZ samples. (B) the clusters expression patterns of upregulated DEGs in HLZ samples. The size indicates the number of unigenes in each cluster.

**Figure 4:**
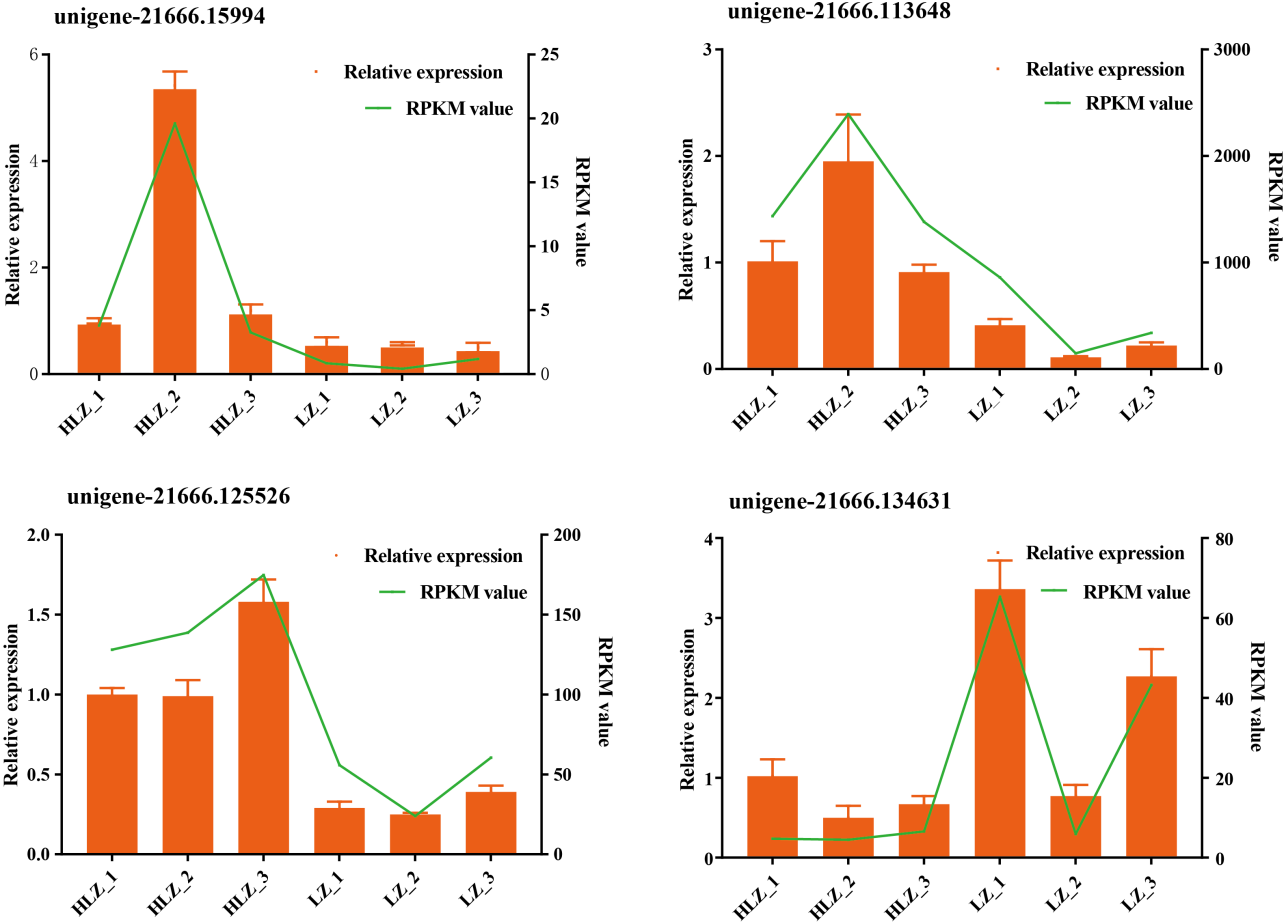
Validation of the RNA-Seq data by qRT-PCR. The green line indicates the RNA-Seq expression data, and the dark yellow bars indicate the qRT-PCR results.

Differential expression analysis between the LZ and HLZ groups revealed 449 differentially expressed genes; approximately 190 DEGs were downregulated in the HLZ group, and 259 DEGs were upregulated in HLZ group. The downregulated genes in HLZ were classified into 10 clusters (Fig. 3A; Table S5). The results showed that cluster 2 included 1 gene (*unigene-21666.123476*) annotated with metallothionein that was highly expressed in all six samples, and cluster 1 included 8 genes that were more highly expressed in LZ samples. *unigene-21666.69211* (mitogen-activated protein kinase kinase kinase ANP1), *unigene-21666.134631* (SAUR family protein), *unigene-21666.128797* (EREBF-like factor), and others without a specific annotation were included. The upregulated genes in HLZ were classified into 10 clusters (Fig. 3B; Table S6), where cluster 5 included 2 genes, and cluster 1 contained 9 genes that might play a vital role, including *unigene-21666.113648* (phenylalanine ammonia-lyase), *unigene-21666.90795* (phenylalanine ammonia-lyase); *unigene-21666.169778* (serine/threonine-protein kinase PBS1), *unigene-21666.167280* (granule-bound starch synthase), *unigene-21666.149531* (acyl-[acyl-carrier-protein] desaturase), *unigene-21666.125526* (anthranilate O-methyltransferase), *unigene-21666.107961* (serine/threonine-protein kinase PBS1), and others without specific annotations (Fig. 3). Random DEGs were chosen for qRT-PCR analysis to validate the accuracy of the RNA-Seq data results. The relative expression results showed a strong correlation between the RNA-Seq and qRT-PCR data (Fig. 4; Table S7).

### Functional classification of all DEGs

**Figure 5:**
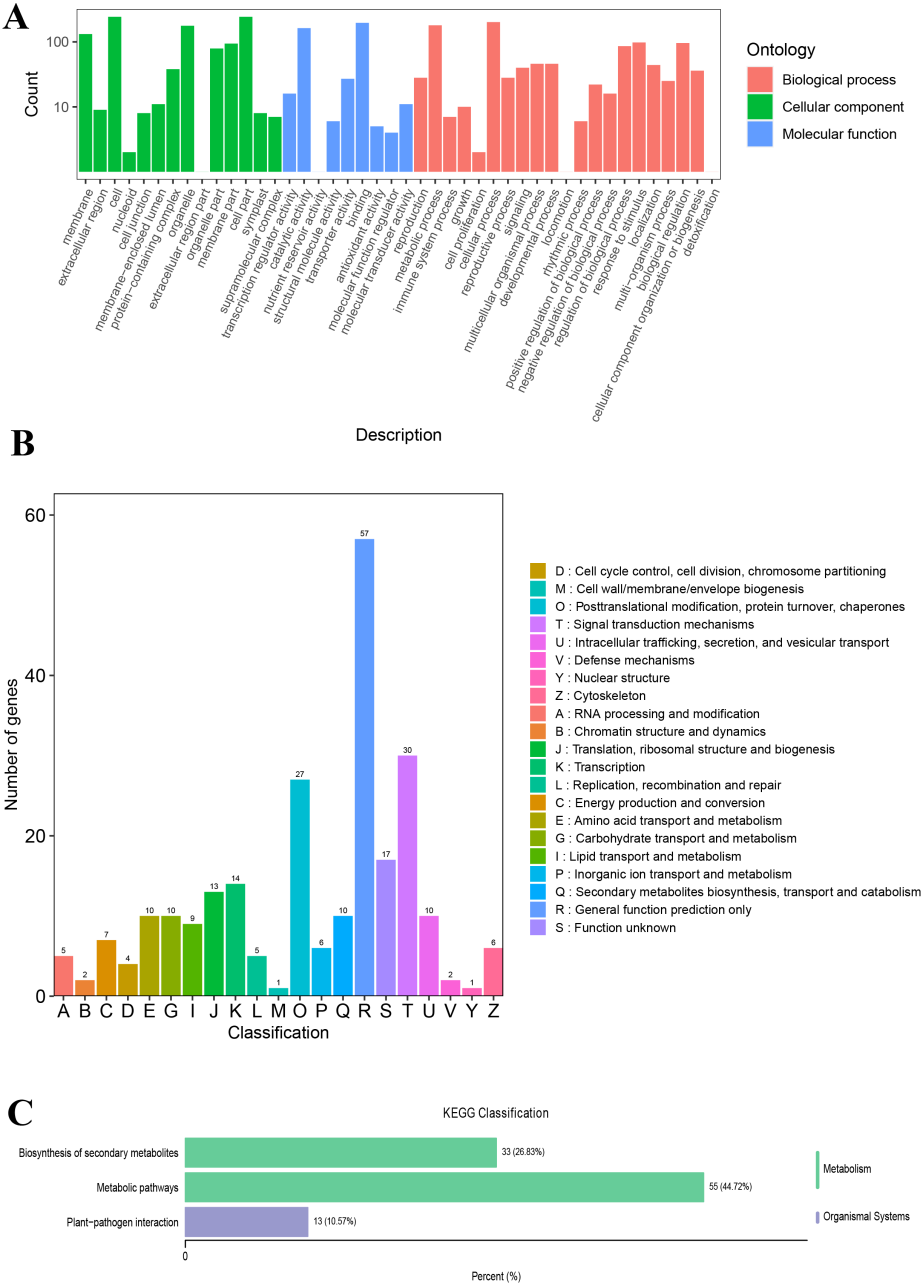
The GO, KOG, and KEGG functional classifications of all DEGs between LZ and HLZ culm skins. (A) The GO functional classification, (B) the KOG functional classification, (C) the KEGG functional classification.

All 449 DEGs were processed for GO, KOG, and KEGG classification. The cellular process (GO:0009987), metabolic process (GO:0008152), response to stimulus (GO:0050896), and biological regulation (GO:0065007) terms were the most common BP ontologies; cell (GO:0005623), cell part (GO:0044464), organelle (GO:0043226), and membrane (GO:0016020) were the most common CC ontologies; and binding (GO:0005488) and catalytic activity (GO:0003824) were the most common MF ontologies (Fig. 5A; Table S8). For the KOG classification, the majority of the DEGs were involved in signal transduction mechanisms, followed by posttranslational modification, protein turnover, chaperones, transcription, translation, ribosomal structure, and biogenesis (Fig. 5B; Table S9). Based on the KEGG classification results, the DEGs were mostly involved in metabolic pathways (ko01100), followed by biosynthesis of secondary metabolites (ko01110) and plant-pathogen interactions (ko04626) (Fig. 5C).

### Functional enrichment of up- and downregulated DEGs

Differential expression analysis between the LZ and HLZ groups showed that 190 and 259 DEGs were down- and upregulated in the HLZ culm skin samples. The GO functional enrichment revealed that the downregulated DEGs were more enriched in monocarboxylic acid transport (GO:0008028), transporter complex (GO:1990351), transmembrane transporter complex (GO:1902495), replication fork (GO:0005657), organelle envelope lumen (GO:0031970), lipid transporter activity (GO:0005319), and monocarboxylic acid transmembrane transporter activity (GO:0008028) (Fig. S1; Table S10).

The upregulated DEGs were more enriched in biological processes and molecular functions. In particular, the top three terms were L-phenylalanine metabolic process (GO:0006558), L-phenylalanine catabolic process (GO:0006559), and entrainment of the circadian clock (GO:0009649), followed by photoreceptor activity (GO:0009881), phenylalanine ammonia-lyase activity (GO:0045548), NAD(P)H dehydrogenase (quinone) activity (GO:0003955), and carbon-nitrogen lyase activity (GO:0016840) (Fig. S2; Table S11).

The downregulated DEGs were enriched in the alpha-linolenic acid metabolism pathway (ko00592) (Fig. S3), and the upregulated DEGs were enriched in the unsaturated fatty acids biosynthesis (ko01040), linoleic acid metabolism (ko00591), biosynthesis of secondary metabolites (ko01110), phenylpropanoid biosynthesis (ko00940), and phenylalanine metabolism (ko00360) pathways (Fig. S4).

### Phytohormones control culm color variation

**Figure 6:**
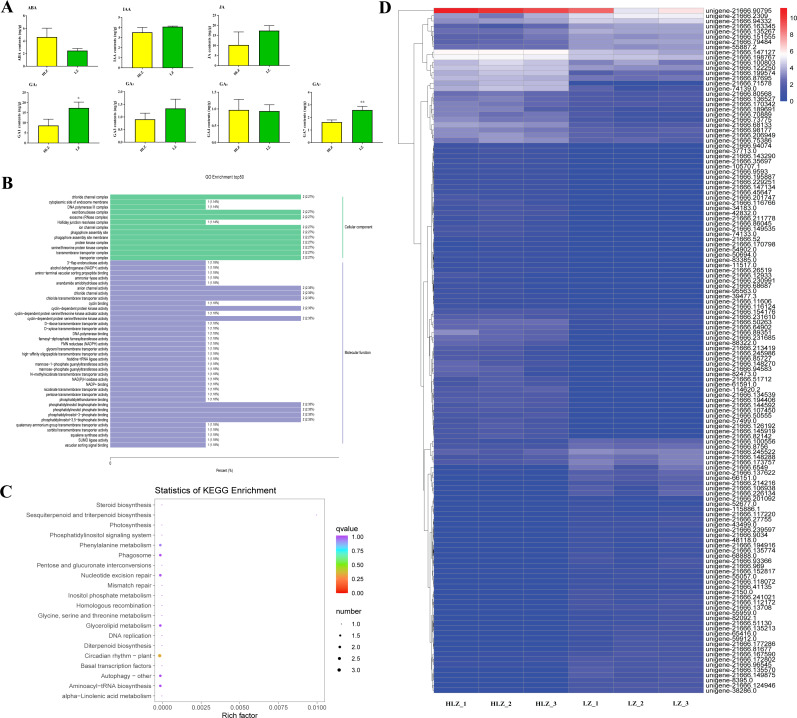
Phytohormones conduct culm color variation in bamboos. (A) The differences in phytohormone contentss between LZ and HLZ, (B) The GO functional enrichment of genes significantly related to ABA, GA_1_, and GA_7_, (C) The KEGG functional enrichment of genes significantly related to ABA, GA_1_, and GA_7_ , (D) The expression heatmap of the genes significantly related to ABA, GA_1_, and GA_7_.

To reveal how phytohormones control culm color variation in *B. oldhamii*, the content of endogenous abscisic acid (ABA), auxin (indole-3-acetic acid, IAA), jasmonic acid (JA), and gibberellic acid (GA_1_, GA_3_, GA_4_, and GA_7_) was detected (Fig. 6A). The majority of phytohormones were highly accumulated in LZ, especially GA_1_ and GA_7_, which were significantly accumulated in LZ; the contents of ABA were relatively highly accumulated in HLZ (Fig. 6A; Table S12). The phytohormones of GA_1_, GA_7_ and ABA were choosen for further study, and the relationship between phytohormones (ABA, GA_1_, and GA_7_) and gene expression was analyzed with corresponding samples. There were 18 genes whose expression was significantly related to ABA accumulation patterns (Table S13), while 35 and 91 genes were significantly related to GA_1_ (Table S14) and GA_7_ (Table S15) accumulation patterns respectively. The genes significantly related to ABA, GA_1_ and GA_7_ were more enriched in the GO terms of chloride channel complex (GO:0034707), ion channel complex (GO:0034702), protein kinase complex (GO:1902911), transporter complex (GO:1990351), anion channel activity (GO:0005253), chloride channel activity (GO:0005254), phosphatidylinositol bisphosphate binding (GO:1902936), and phosphatidylinositol-3,5-bisphosphate binding (GO:0080025) (Fig. 6B; Table S16). All the genes significantly related to ABA, GA_1_ and GA_7_ were also enriched in the circadian rhythm (plant) pathway (ko04712) (Fig. 6C; Table S17). In the circadian rhythm (plant) pathway, the bZIP transcription factor HY5 (*unigene-21666.126192* and *unigene-21666.239597*) and protein FLOWERING LOCUS T (*FT*) (*unigene-54902.0*) were up-regulated. Moreover, the expression of genes significantly related to ABA, GA_1_ and GA_7_ showed that *unigene-21666.90795* (*phenylalanine/tyrosine ammonia-lyase*, *PTAL*) was relatively highly expressed in HLZ (Fig. 6D). The gene of *PTAL* is a crucial gene in phenylalanine metabolism and phenylpropanoid biosynthesis pathway which might conduct bamboo culm color variation.

### Differentially expressed transcription factors

**Figure 7:**
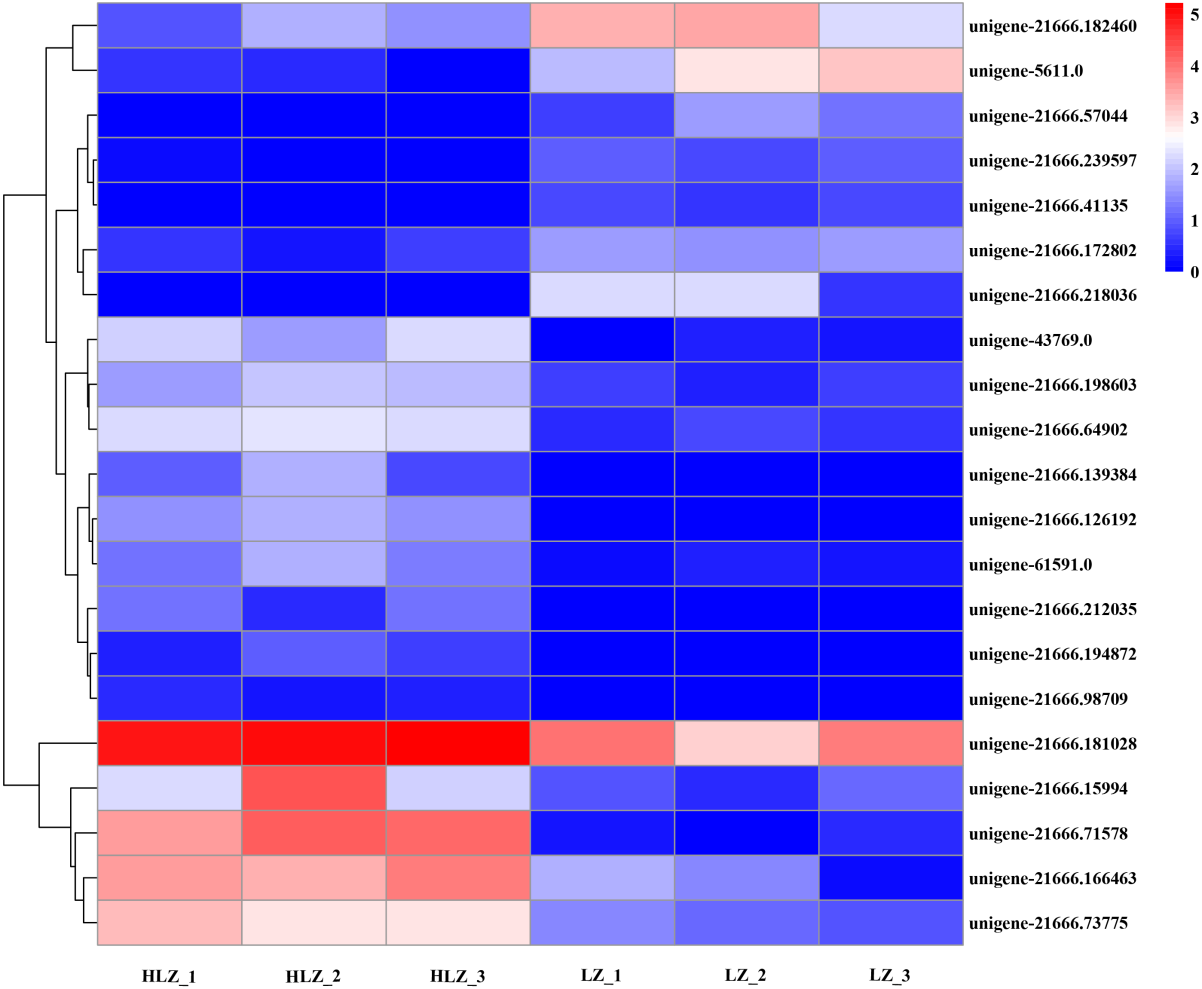
Differentially expressed transcription factors between LZ and HLZ.

Differentially expressed transcription factors were filtered out of the annotated unigenes. A total of 21 differentially expressed transcription factors were identified (Fig. 7; Table S18). The genes *unigene-21666.57044* (bHLH), *unigene-21666.218036* (MYB-related), and *unigene-21666.41135* (SBP) were not detected in the HLZ samples and were only detected in the LZ samples. bHLH transcription factors can interact with MYB transcription factors to regulate anthocyanin biosynthesis (Hichri et al., 2010). However, *unigene-21666.194872* (FAR1), *unigene-21666.98709* (MADS-M-type), *unigene-21666.139384* (MYB), and *unigene-21666.212035* (TRAF) were not detected in the LZ samples and were only detected in the HLZ samples. MADS-box transcription factors are involved in flower promotion and development, and simultaneous death usually follows after mass production of bamboo flowers (Abe, Miguchi & Nakashizuka, 2001). Four genes belonged to the bZIP transcription factor family, among which three genes (*unigene-21666.126192*, *unigene-21666.239597*, and *unigene-43769.0*) were annotated as the transcription factor HY5. The target genes of HY5 participate in many biological signaling processes, such as light signaling, circadian clock, anthocyanin biosynthesis, and chlorophyll biosynthesis (Gangappa & Botto, 2016). HY5 could induce the expression of the structural genes *CHS* (chalcone synthase), *CHI* (chalcone isomerase), and *FLS* (flavonol synthase) to regulate anthocyanin biosynthesis (Gangappa & Botto, 2016). There were three genes (*unigene-21666.139348*, *unigene-21666.15994*, and *unigene-61591.0*) that were members of the MYB transcription factor family. MYB transcription factors could regulate anthocyanin biosynthesis (Wang et al., 2019) and combine with other transcription factors to form MYB-bHLH-WDR complexes to regulate flavonoid biosynthesis (Xu, Dubos & Lepiniec, 2015).

## Discussion

Chlorophyll is a natural green pigment, and during green plant senescence, chlorophyll breakdown leads to a decrease in green color (Hörtensteiner, 2009). In the ripening phase of many fruits, such as tomato and banana, the color variation is caused by the massive degradation of chlorophyll. The *unigene-21666.231610* gene is upregulated in the photosynthesis pathway and was annotated as psbP (photosystem II oxygen-evolving enhancer protein 2). The psbP protein is required for the photosystem II complex and normal thylakoid architecture in *Arabidopsis thaliana* (Yi et al., 2007; Yi et al., 2009). The *unigene-54902.0* (*FT*) gene is upregulated in the circadian rhythm (plant) pathway. The *FT* gene (FLOWERING LOCUS T), a mobile stimulus expressed in leaves and then translocated to the shoot apex, is essential for floral induction in Arabidopsis (Liu, Zhang & Yu, 2020). The MADS-box transcription factor *unigene-21666.98709* (MADS-M-type) is relatively highly expressed in HLZ. MADS-box genes are essential for flower induction, promotion, and maturation (Theißen, 2001). The reproductive cycle of bamboo varies from 3 to 120 years (Janzen, 1976). Mass flowering in some bamboos is usually followed by simultaneous death at some levels (Abe, Miguchi & Nakashizuka, 2001; Miyazaki et al., 2009). Flowering is a hallmark event in the bamboo life cycle, followed by senescence (Marchesini, Sala & Austin, 2009). The decreased chlorophyll contents and increased FT and MADS-box gene expression levels revealed that the culm color variation of *B. oldhamii* might be related to the bamboo flowering trends.

ABA contents were higher in HLZ with green and yellow striped culms in our study. Exogenous ABA could promote the accumulation of anthocyanins in Lycium fruits, and the structural genes involved in the flavonoid biosynthetic pathway were upregulated by ABA treatment (Li et al., 2019). The application of ABA could influence the expression of R2R3 MYB and the bHLH family (Li et al., 2019). The transcription of structural anthocyanin biosynthesis genes is regulated by MYB-bHLH-WD40 complexes (Jaakola, 2013). In *Prunus avium L.*, ABA treatment could influence the expression of PacMYBA and induce anthocyanin accumulation (Shen et al., 2014). The GA_1_ and GA_7_ contents were significantly higher in LZ with green culms. Gibberellins delayed both chlorophyll depletion and total carotenoid accumulation (Alós et al., 2006). GA_1_ and GA _7_ delayed fruit coloration in the flavedo of ‘Washington’ navel sweet orange when girding the fruit peduncle (Gambetta et al., 2012). GA_3_ delayed flavedo chlorophyll degradation and delayed fruit color break by reducing *β*-cryptoxanthin and *β*-citraurin biosynthesis (Gambetta et al., 2014).

The bZIP transcription factor *HY5* can transmit blue light signaling to the circadian rhythm pathway by binding to the promoter of circadian regulated genes (Lee et al., 2007; Hajdu et al., 2018). In Arabidopsis, circadian rhythm pathway genes can synchronize light signals to regulate reproductive growth (Andronis et al., 2008). The *HY5* gene not only regulates light signaling and circadian clock pathways but also regulates anthocyanin biosynthesis, chlorophyll biosynthesis, and hormone signaling pathways (Gangappa & Botto, 2016). Carotenoids produce red, orange, and yellow colors in plants (Stanley & Yuan, 2019). The hormone ABA needs carotenoids as precursors in its synthesis (Fang et al., 2008). HY5 binds to the promoter of *ABI5* and mediates ABA responses (Chen et al., 2008). HY5 also binds to the promoter of the R2R3-MYB transcription factor PAP1 to regulate anthocyanin biosynthesis. Three unigenes were annotated as HY5, among which *unigene-21666.126192* and *unigene-43769.0* were relatively more highly expressed in HLZ, and *unigene-21666.239597* was relatively more highly expressed in LZ. These results indicated that bZIP transcription factors play important roles in bamboo culm color variation.

MYB transcription factors with a highly conserved DNA-binding domain consist of four imperfect amino acid sequence repeats (R). These proteins are usually divided into four classes: (1) R2R3-MYB, (2) 1R-MYB, MYB-related, and others, (3) 3R-MYB, and (4) 4R-MYB (Dubos et al., 2010). Among the plant MYB transcription factors, R2R3-MYB is the most common and is involved in the regulation of flavonoid biosynthesis (Chen et al., 2006; Stracke et al., 2007). Flavonoid-based pigments are produced in the phenylpropanoid pathway. MYB transcription factors can interact with other transcription factors or proteins to form a complex to regulate the phenylpropanoid pathway (Morita et al., 2006). Three MYB genes were highly expressed in HLZ culm skin samples, and the MYB-related gene *unigene-21666.218036* is relatively highly expressed in LZ samples. In kiwifruit, AcMYB123 and AcHLH42 can interact with each other to activate anthocyanin biosynthesis genes (Wang et al., 2019).

The family of Basic Helix-Loop-Helix (bHLH) transcription factors contains approximately 60 conserved amino acid domains that bind to the promoter of E-box cis-elements to regulate downstream genes (Toledo-Ortiz, Huq & Quail, 2003; Hichri et al., 2011), and they play multiple roles in plant development (Hichri et al., 2011). In Arabidopsis, bHLH proteins are involved in flavonoid metabolism and modify seed pigmentation (Nesi et al., 2000). In grapevine, the bHLH transcription factor MYC1 can physically interact with MYB5a, MYB5b, MYBA1/A2, and MYBPA1 to induce transcription from the promoters of flavonoid pathway genes involved in anthocyanin and proanthocyanidin (PA) synthesis (Hichri et al., 2010). Flavonoid biosynthesis is controlled by MYB-bHLH-WDR complexes and is regulated by hormones, the environment, and development (Xu, Dubos & Lepiniec, 2015). Only one bHLH transcription factor, *unigene-21666.57044,* was differentially expressed. These results demonstrate that MYB and bHLH transcription factors could regulate the color variation of bamboos. However, how the crosstalk among the transcription factors and hormone regulation influences color variation still needs further investigation.

## Conclusions

This paper focused on investigating the culm color variation of the clumping bamboo *B. oldhamii* via combined RNA-seq and endogenous phytohormone content variation analyses. The results showed that bZIP, MYB, HY5, and other differentially expressed transcription factors play a role in *B. oldhamii* culm color variation. Moreover, phytohormone contents, especially GA_1_ and GA _7,_ were more highly accumulated in LZ, but many flower-regulated genes were more highly expressed in HLZ, which indicates that HLZ may flower more rapidly than LZ and that the senescence pathways may be involved in bamboo culm color variation. The transcription factors HY5, MYB, and bHLH participate in culm color variation by regulating pigment biosynthesis pathways to cause bamboo culm color variation, but how the regulatory pathways between transcription factors and phytohormones influence culm color variation still needs to be deeply investigated.

## Supplemental Information

10.7717/peerj.12796/supp-1 Supplemental Information 1 GO enrichment of downregulated DEGs

10.7717/peerj.12796/supp-2 Supplemental Information 2 GO enrichment of upregulated DEGs

10.7717/peerj.12796/supp-3 Supplemental Information 3 KEGG enrichment of downregulated DEGs

10.7717/peerj.12796/supp-4 Supplemental Information 4 KEGG enrichment of upregulated DEGs

10.7717/peerj.12796/supp-5 Supplemental Information 5 Sequences of the primers used in the qRT-PCR experiment

10.7717/peerj.12796/supp-6 Supplemental Information 6 The GO classification of all unigenes

10.7717/peerj.12796/supp-7 Supplemental Information 7 The KOG classification of all unigenes

10.7717/peerj.12796/supp-8 Supplemental Information 8 KEGG pathway classification of all unigenes

10.7717/peerj.12796/supp-9 Supplemental Information 9 The ten clusters expression pattern of downregulated DEGs

10.7717/peerj.12796/supp-10 Supplemental Information 10 The ten clusters expression pattern of upregulated DEGs

10.7717/peerj.12796/supp-11 Supplemental Information 11 The FPKM values and relative expression of genes for qRT-PCR

10.7717/peerj.12796/supp-12 Supplemental Information 12 The GO classification of all DEGs

10.7717/peerj.12796/supp-13 Supplemental Information 13 The KOG classification of all DEGs

10.7717/peerj.12796/supp-14 Supplemental Information 14 The GO functional enrichment of down-regulated DEGs

10.7717/peerj.12796/supp-15 Supplemental Information 15 The GO functional enrichment of up-regulated DEGs

10.7717/peerj.12796/supp-16 Supplemental Information 16 The contents of phytohormones between LZ and HLZ

10.7717/peerj.12796/supp-17 Supplemental Information 17 The significantly related Unigenes of ABA contents between LZ and HLZ

10.7717/peerj.12796/supp-18 Supplemental Information 18 The significantly related Unigenes of GA_1_ contents between LZ and HLZ

10.7717/peerj.12796/supp-19 Supplemental Information 19 The significantly related Unigenes of GA_7_ contents between LZ and HLZ

10.7717/peerj.12796/supp-20 Supplemental Information 20 The GO functional enrichment of genes significantly related to ABA, GA_1_, and GA_7_

10.7717/peerj.12796/supp-21 Supplemental Information 21 The KEGG functional enrichment of genes significantly related to ABA, GA_1_, and GA_7_

10.7717/peerj.12796/supp-22 Supplemental Information 22 The FPKM values of differentially expressed transcription factors between LZ and HLZ culm skins

## Additional Information and Declarations

### Data Availability

The following information was supplied regarding data availability: The data are available at the SRA: PRJNA706800; HLZ_1, SAMN18145072 and SRR13860619; HLZ_2, SAMN18145073 and SRR13860618; HLZ_3, SAMN18145074 and SRR13860617; LZ_1, SAMN18145075 and SRR13860616; LZ_2, SAMN18145076 and SRR13860615; LZ_3, SAMN18145077 and SRR13860614. This Transcriptome Shotgun Assembly project is available at DDBJ/EMBL/GenBank: GJPT00000000. The version described in this article is the first version, GJPT01000000. The annotation of all unigenes is available at FigShare: Jiao, Yulian; Zeng, Hu; Xia, Haitao; Wang, Yueying; Wang, Jinwang; Jin, Chuan (2021): All 383278 Unigenes annotation of Bambusa oldhamii Munro culm skin transcriptome. figshare. Dataset. https://doi.org/10.6084/m9.figshare.16912324.v3.
