# Supplementary figures and images for: RNA-seq and phytohormone analysis reveals the culm color variation of Bambusa oldhamii Munro
Source: PeerJ. 2022 Jan 13;10:e12796. doi: 10.7717/peerj.12796 (PMC8761368; doi:10.7717/peerj.12796)

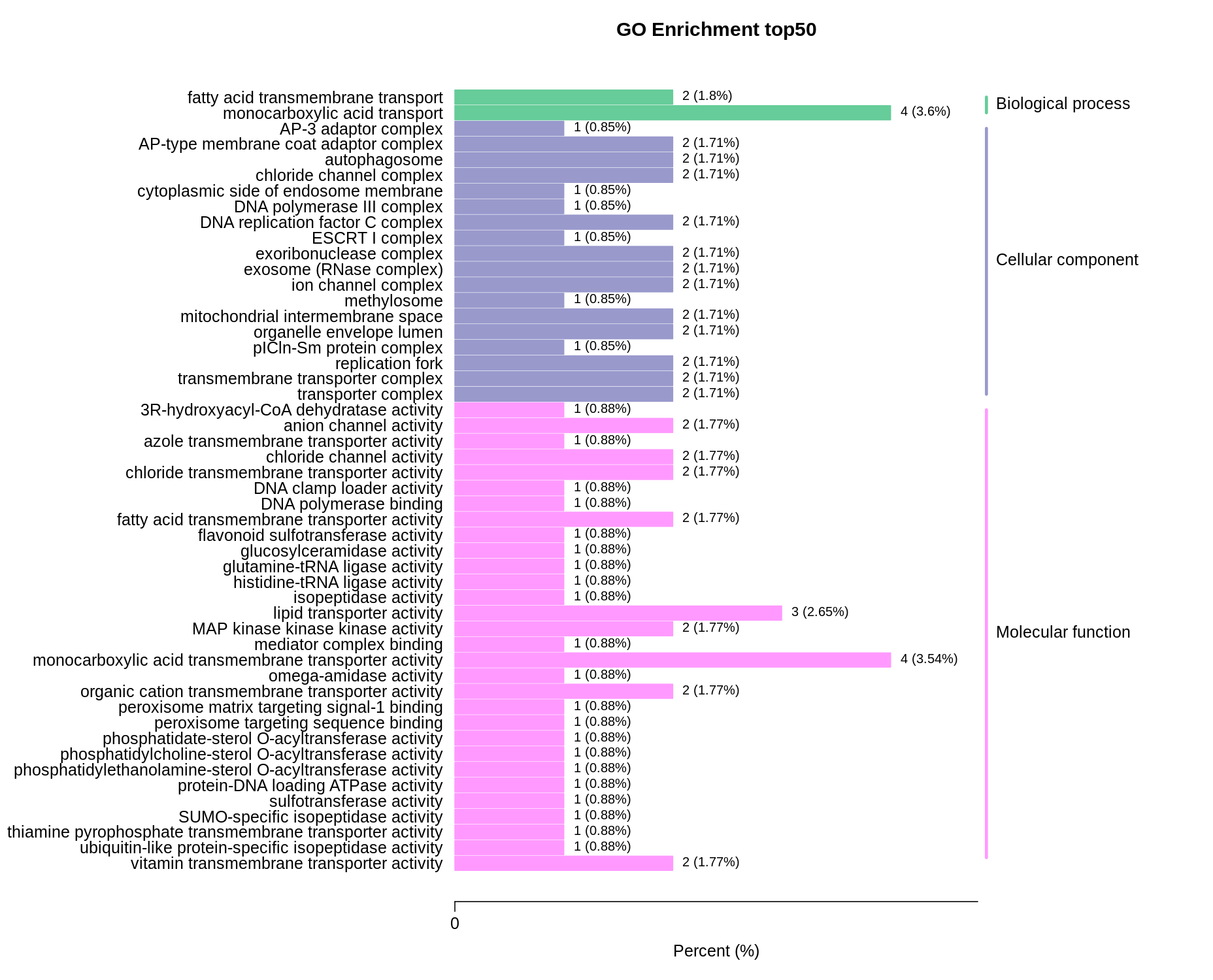

Supplement: Supplemental Information 1 [file peerj-10-12796-s001.png]

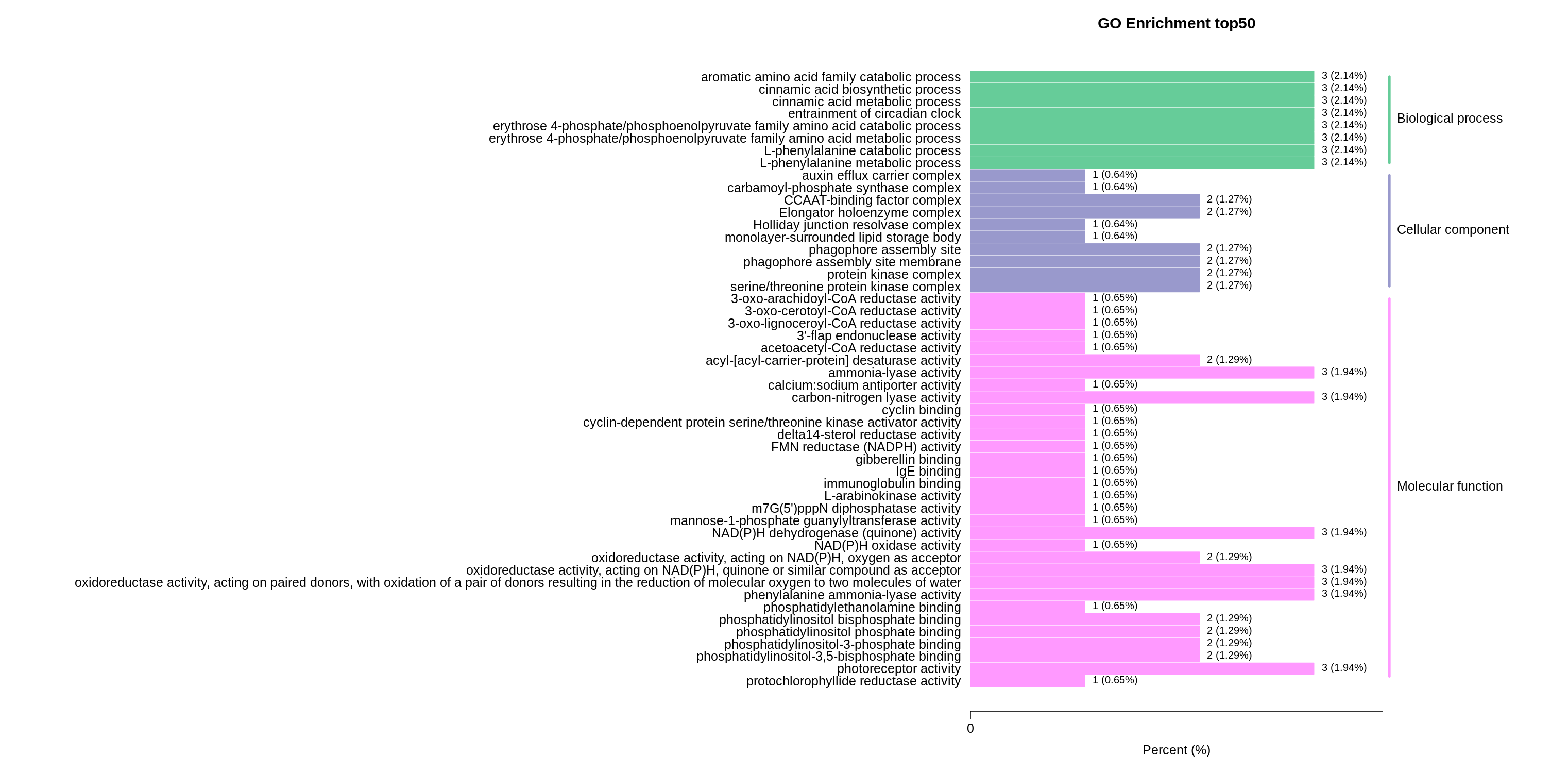

Supplement: Supplemental Information 2 [file peerj-10-12796-s002.png]

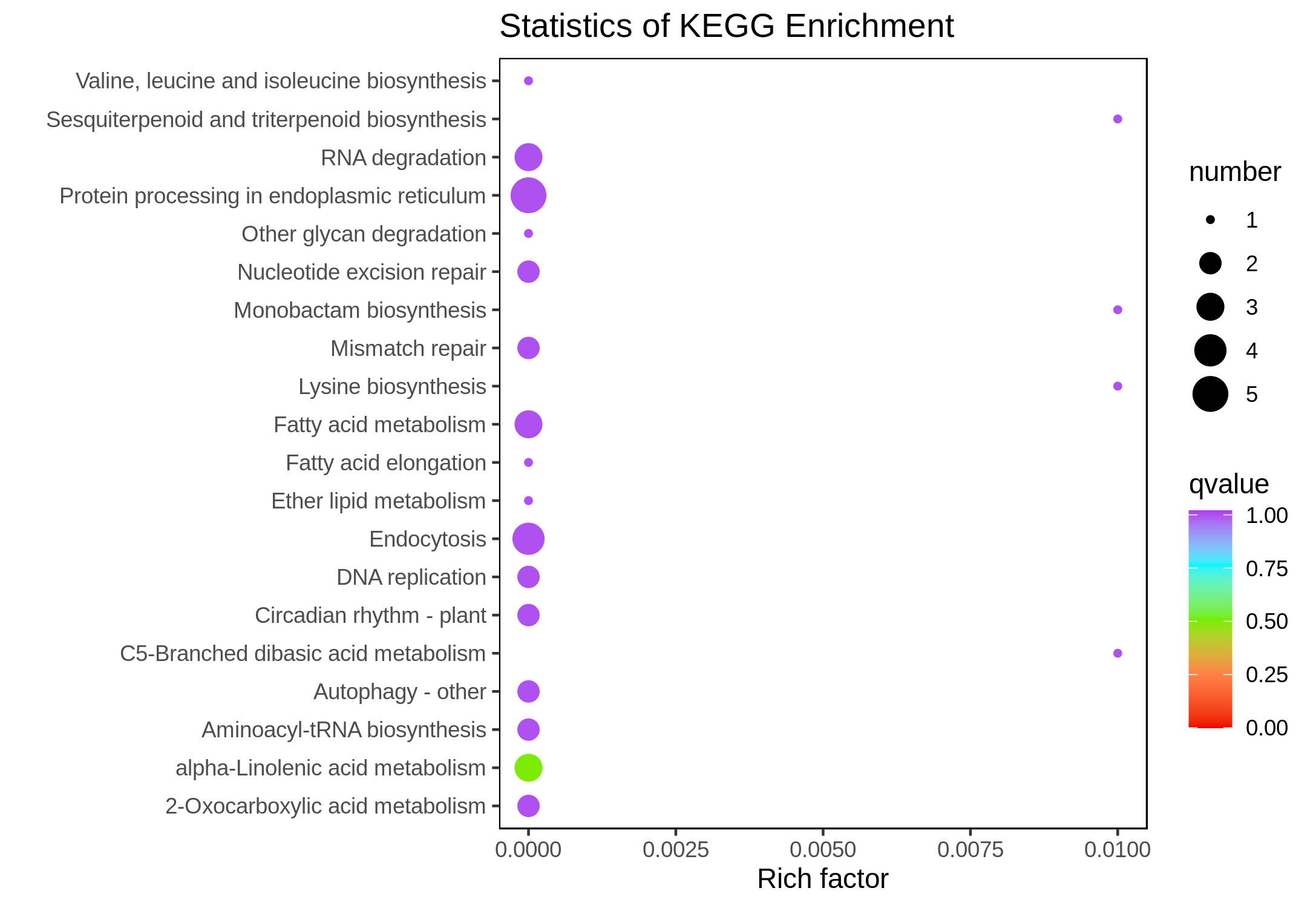

Supplement: Supplemental Information 3 [file peerj-10-12796-s003.png]

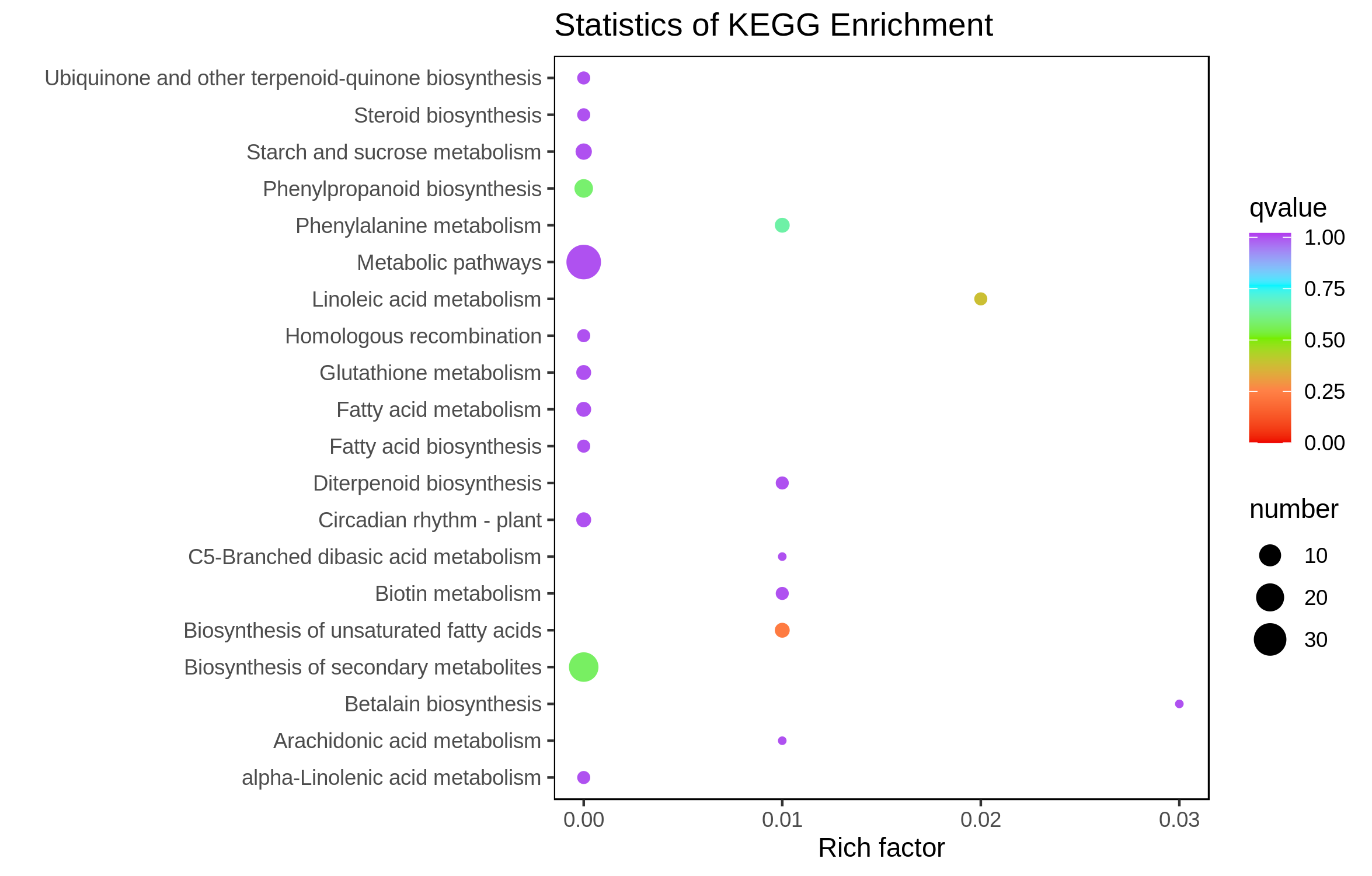

Supplement: Supplemental Information 4 [file peerj-10-12796-s004.png]
